# Mutant U2AF1-expressing cells are sensitive to pharmacological modulation of the spliceosome

**DOI:** 10.1038/ncomms14060

**Published:** 2017-01-09

**Authors:** Cara Lunn Shirai, Brian S. White, Manorama Tripathi, Roberto Tapia, James N. Ley, Matthew Ndonwi, Sanghyun Kim, Jin Shao, Alexa Carver, Borja Saez, Robert S. Fulton, Catrina Fronick, Michelle O'Laughlin, Chandraiah Lagisetti, Thomas R. Webb, Timothy A. Graubert, Matthew J. Walter

**Affiliations:** 1 Division of Oncology, Washington University School of Medicine, St Louis, Missouri 63110, USA; 2 Massachusetts General Hospital Cancer Center, Boston, Massachusetts 02114, USA; 3 McDonnell Genome Institute, Washington University, St Louis, Missouri 63108, USA; 4 SRI International, Bioscience Division, Menlo Park, California 94025, USA

## Abstract

Somatic mutations in spliceosome genes are detectable in ∼50% of patients with myelodysplastic syndromes (MDS). We hypothesize that cells harbouring spliceosome gene mutations have increased sensitivity to pharmacological perturbation of the spliceosome. We focus on mutant U2AF1 and utilize sudemycin compounds that modulate pre-mRNA splicing. We find that haematopoietic cells expressing mutant U2AF1(S34F), including primary patient cells, have an increased sensitivity to *in vitro* sudemycin treatment relative to controls. *In vivo* sudemycin treatment of U2AF1(S34F) transgenic mice alters splicing and reverts haematopoietic progenitor cell expansion induced by mutant U2AF1 expression. The splicing effects of sudemycin and U2AF1(S34F) can be cumulative in cells exposed to both perturbations—drug and mutation—compared with cells exposed to either alone. These cumulative effects may result in downstream phenotypic consequences in sudemycin-treated mutant cells. Taken together, these data suggest a potential for treating haematological cancers harbouring *U2AF1* mutations with pre-mRNA splicing modulators like sudemycins.

## Abstract (web summary)

Spliceosome mutations occur in approximately 50% of patients with myelodysplastic syndromes. Here, the authors show that tumour cells harbouring the *S34F* mutation in the *U2AF* spliceosome gene is sensitive to compounds that further perturb the spliceosome.

Myelodysplastic syndromes (MDS) are the most common adult myeloid malignancy with up to 40,000 new cases diagnosed each year in the United States^1^,^2^. MDS are a heterogeneous group of clonal haematopoietic stem cell disorders characterized by peripheral blood cytopaenias and progenitor expansion; approximately one-third of patients will transform to a secondary acute myeloid leukaemia (AML) that has a poor prognosis^3^. The only curative therapy is bone marrow transplantation, which is often not an option because of patient comorbidities^3^. New treatment approaches are greatly needed. At least half of all MDS patient bone marrow samples harbour a mutation in one of several spliceosome genes^4^,^5^,^6^,^7^,^8^,^9^,^10^, highlighting a potential genetic vulnerability. In addition, spliceosome gene mutations often occur in the founding clones of MDS tumours, providing an attractive target for elimination of all tumour cells^10^,^11^. Spliceosome gene mutations are mutually exclusive of each other in patients^4^,^10^,^11^,^12^, implying either a redundancy in pathogenic function or that a cell cannot tolerate two spliceosome perturbations at once. With this in mind, we hypothesized that cells harbouring a spliceosome gene mutation would have increased sensitivity to further perturbation of the spliceosome by splicing modulator drugs. To examine this, we utilized sudemycin compounds that bind the SF3B1 spliceosome protein and modulate pre-mRNA splicing^13^,^14^,^15^. We used sudemycin D1 and D6, which are synthetic compounds that have been optimized by several rounds of medicinal chemistry for their potent *in vivo* antitumour activity^13^. We examined the sensitivity of spliceosome mutant cells to sudemycin treatment, focusing on mutations in the spliceosome gene *U2AF1*, which have been identified in 11% of MDS patients, utilizing the S34F missense mutation most commonly found in our studies^4^,^5^. Mutant U2AF1(S34F) expression has been shown by our group and others to cause altered pre-mRNA splicing in a variety of cell types, as well as altered haematopoiesis and pre-mRNA splicing in mice^4^,^5^,^16^,^17^,^18^,^19^.

In this manuscript, we provide evidence that U2AF1(S34F)-expressing cells are sensitive to the splicing modulator drug sudemycin. Haematopoietic cells expressing mutant U2AF1 show reduced survival and altered cell cycle in response to sudemycin D6 *in vitro*. *In vivo* treatment of U2AF1(S34F) transgenic mice with sudemycin results in an attenuation of mutant U2AF1-induced haematopoietic progenitor cell expansion that is associated with increased cell death. In addition, unsupervised analysis of whole-transcriptome sequencing (RNA-seq) finds that sudemycin D6 perturbs RNA splicing in both mutant U2AF1(S34F)- and U2AF1(WT)-expressing bone marrow cells; however, sudemycin D6 treatment further modulates mutant U2AF1(S34F)-induced splicing changes to create cumulative effects on cells *in vivo*. The cumulative RNA-splicing effects of sudemycin and mutant U2AF1 may contribute to the downstream phenotypic consequences we observe *in vivo*.

## Results

### Sudemycin alters RNA splicing in primary human CD34+ cells

**Figure 1:**
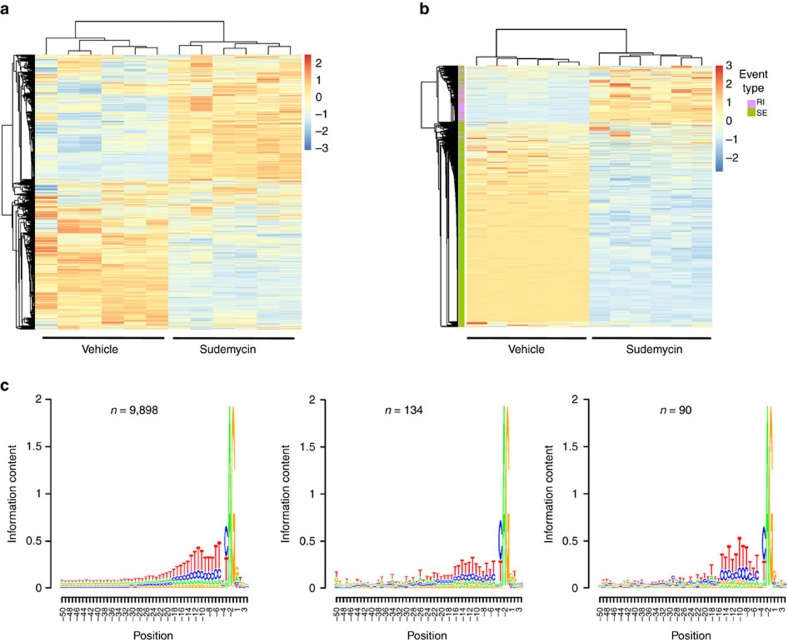
Sudemycin D6 alters gene expression and pre-mRNA splicing in primary human CD34+ haematopoietic cells. Whole-transcriptome (that is, RNA-seq) analysis was performed on CD34+ cells isolated from human umbilical cord blood following treatment of samples with 1,000 nM Sudemycin D6 or DMSO vehicle for 6 h (*n*=6). Unsupervised hierarchical clustering of (**a**) expressed genes and (**b**) splice junctions. Skipped exons (SE, green) and retained introns (RI, purple) event types are visualized. Values are *z*-scores computed from regularized logarithm values for genes and from per cent spliced in (PSI or Ψ) values for splicing events. (**c**) Intronic sequence contexts of cassette exon 3′ splice sites skipped more often in sudemycin- relative to vehicle-treated cells (FDR<5%, |ΔΨ|>10%, left panel) or skipped more often in vehicle-relative to sudemycin-treated cells (FDR<5%, |ΔΨ|>10%, middle panel), along with a context of unperturbed control exons (FDR>50%, |ΔΨ|<0.1%, right panel). Position is relative to the first base in the exon.

We first examined the pre-mRNA splicing alterations induced by sudemycin D6 in primary human haematopoietic cells. We treated CD34+ haematopoietic progenitor cells isolated from human umbilical cord blood with 1,000 nM of sudemycin D6 or dimethylsulphoxide (DMSO) vehicle control for 6 h *in vitro* and performed whole-transcriptome (RNA-seq) analysis (*n*=6 each, Supplementary Fig. 1). We identified robustly altered gene expression and pre-mRNA splicing patterns induced by sudemycin, as shown by unsupervised clustering of samples using expressed genes (Fig. 1a) and pre-mRNA splice junctions (Fig. 1b), respectively. Our analysis identified 1,030 differentially expressed genes (FDR<5%, |log_2_FC|>1) and 18,833 dysregulated splicing events (FDR<5%, |delta per cent spliced in or PSI (ΔΨ)|>10%, Supplementary Data 1 and 2, respectively) that discriminated between sudemycin D6-treated samples and controls. Sudemycin D6 treatment induced altered pre-mRNA splicing with a bias towards increased exon skipping and intron retention (Fig. 1b). However, there was no apparent bias in the sequence motif surrounding splice acceptor sites of cassette exons that were alternatively spliced (Fig. 1c), in contrast to previously observed biases in sequences surrounding alternatively spliced junctions induced by expression of mutant spliceosome proteins U2AF1, SF3B1 and SRSF2 (refs 16, 17, 18, 19, 20, 21, 22, 23, 24, 25).

To determine whether particular pathways are enriched for splicing perturbations, we applied GOseq to 6,278 genes with junctions significantly altered by sudemycin D6 treatment (FDR<5%, |log_2_FC|>2). While pathway enrichment was minimal (enrichment scores <2), GOseq analysis indicated that pathways involved in pre-mRNA splicing, RNA processing and transport, cell cycle, as well as ATPase and helicase activity were enriched in splice junctions altered by sudemycin D6 treatment (FDR<10%; Supplementary Data 3). Genes with sudemycin-altered expression were enriched in pathways involved in receptor and signal transduction activities (FDR<10%; Supplementary Data 4).

### Mutant U2AF1 cells have increased sensitivity to sudemycin

**Figure 2:**
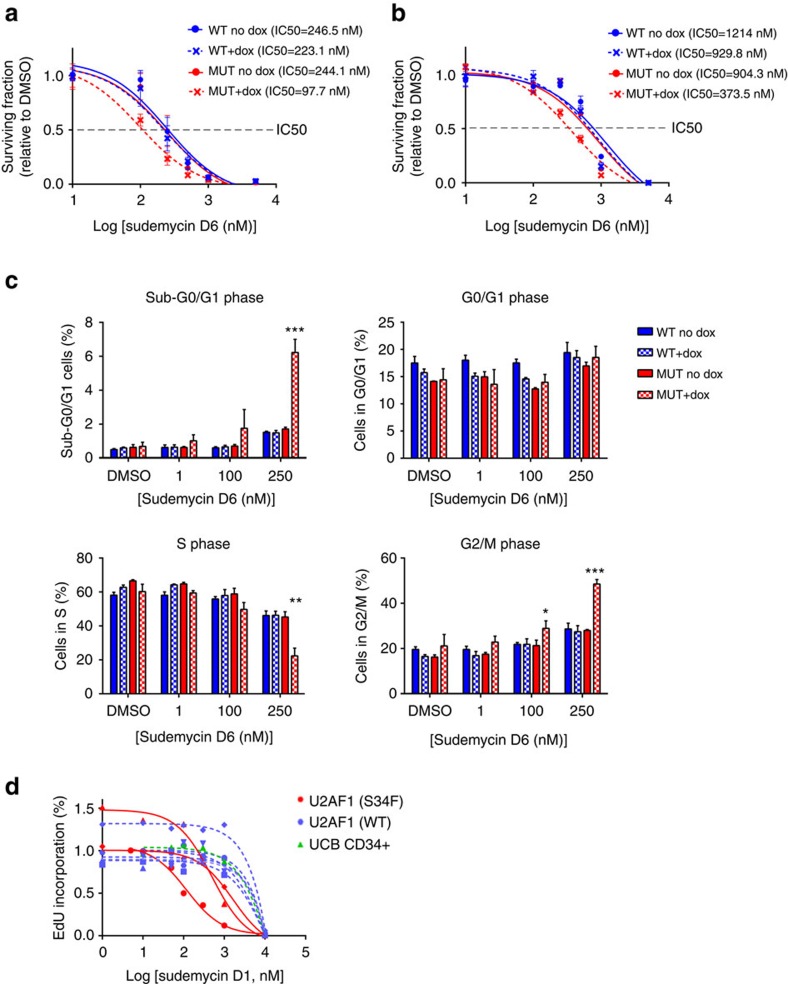
Mutant U2AF1(S34F)-expressing cells display increased sensitivity to sudemycin D *in vitro.* (**a**) K562 cells (*n*=6 for control groups, *n*=9 for U2AF1(S34F) treated with doxycycline) or (**b**) OCI-AML3 cells (*n*=3 for all groups) with stably integrated, doxycycline-inducible U2AF1(WT) or mutant U2AF1(S34F) were cultured with increasing concentrations of sudemycin D6 concurrently with doxycycline (250 ng ml^−1^, where indicated) for 5 days following 2 days of initial induction of mutant or WT U2AF1; total cell numbers were measured. The surviving fraction of cells is shown. IC50, inhibitory concentration at 50% of maximum cell survival. (**c**) K562 cell cycle phases were determined using BrdU/7AAD (*n*=3, representative of two experiments; *P<0.05, **P<0.01, ***P<0.001 statistics calculated with two-tailed *t-*tests of MUT + dox samples compared with each control group at a given concentration of sudemycin D6, and the least significant value is given for each group; mean values with s.d. shown). (**d**) Primary human MDS or AML cells (both mutant U2AF1(S34F) samples (*n*=3) and those wild type for *U2AF1* (*n*=6)) or normal umbilical cord blood CD34+ cells (*n*=1) were cultured on irradiated HS27 stroma, and proliferation (EdU incorporation) was measured after 3 days of exposure to increasing concentrations of sudemycin D1.

To examine the effects of sudemycin D6 on haematopoietic cells expressing mutant U2AF1, we generated K562 human erythroleukaemia and OCI-AML3 AML cell lines that have stably integrated doxycycline-inducible, FLAG-tagged U2AF1(S34F) or FLAG-tagged U2AF1(WT) to control for U2AF1 overexpression (Supplementary Fig. 2a,b for K562; Fig. 2c,d for OCI-AML3). Mutant U2AF1(S34F)-expressing K562 cells showed reduced survival and lower IC50 (*P*<0.0001, extra sum-of-squares F-test) relative to uninduced mutant U2AF1(S34F) and U2AF1(WT)-expressing control cells (Fig. 2a). These effects were also observed in human OCI-AML3 cell lines expressing mutant U2AF1(S34F) compared with U2AF1(WT)-expressing cells and other control cells (*P*<0.003, extra sum-of-squares F-test; Fig. 2b). Reduced survival of K562 cells in the presence of sudemycin D6 is associated with an altered cell cycle profile: U2AF1(S34F)-expressing K562 cells had a decrease of cells in the S-phase and an increase of cells in the sub-G0/G1 and G2/M phases (Fig. 2c). Furthermore, MDS or AML cells with U2AF1(S34F) mutations treated *in vitro* with sudemycin D1, a sudemycin compound very similar to D6, showed an increased sensitivity to sudemycin (reduced S-phase) relative to control MDS/AML cells without spliceosome gene mutations (Fig. 2d). In contrast, treatment of MDS/AML patient cells with the chemotherapeutic drug daunorubicin (not predicted to disrupt splicing) showed no specificity for mutant U2AF1(S34F) samples compared with controls (Supplementary Fig. 2e). In addition, human CD34+ cells expressing U2AF1(S34F) showed increased sensitivity to another splicing modulator drug (E7107) similar to sudemycin (Supplementary Fig. 2f).

### Sudemycin reduces mutant U2AF1 progenitor expansion *in vivo*

**Figure 3:**
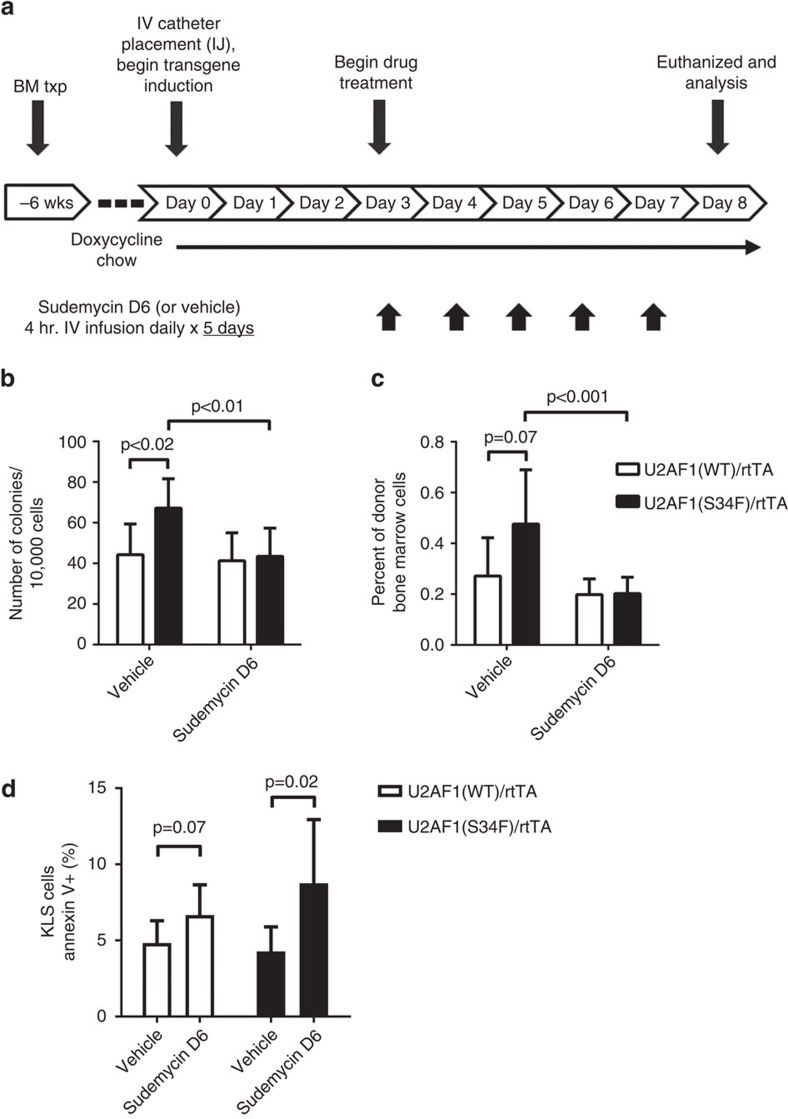
Sudemycin D6 treatment attenuates mutant U2AF1-induced progenitor cell expansion in U2AF1(S34F) transgenic mice. (**a**) Schema of sudemycin treatment of transgenic mice *in vivo*. Doxycycline-inducible U2AF1(S34F) or U2AF1(WT) transgenic mouse bone marrow was transplanted into recipient mice. Intrajugular (IJ) catheters were placed for IV drug infusions, which were performed over 4 h for 5 days. (**b**) Haematopoietic progenitor CFU-C colony-forming assay and (**c**) flow cytometry for haematopoietic stem and progenitor (HSPC) cell surface markers (c-Kit+, lineage−, Sca1+, KLS, right panel) on U2AF1(WT)- or (S34F) mutant-recipient mouse bone marrow following treatment is shown (*n*=6 for both U2AF1(WT) conditions, *n*=7 for vehicle-treated U2AF1(S34F), *n*=11 for sudemycin D6-treated U2AF1(S34F)). (**d**) Annexin V+ KLS cells were quantified in the bone marrow of mice following *in vivo* sudemycin treatment as described above (*n*=7–9). Data pooled from two independent experiments (**b**–**d**); statistics calculated using two-tailed *t*-tests for each comparison shown; mean values with s.d. shown.

We next examined the effect of sudemycin treatment *in vivo* on mutant U2AF1(S34F)-induced phenotypes using our previously described U2AF1(S34F) transgenic mouse model^19^. We induced U2AF1(S34F) or U2AF1(WT) transgenes for 7 days in the bone marrow cells of transplanted mice (to study haematopoietic cell-intrinsic effects) and treated mice concurrently with sudemycin D6 (50 mg kg^−1^ per day) or vehicle for 5 of those days; see schema (Fig. 3a). Sudemycin D6 treatment of transplanted mice showed an attenuation of the previously described^19^ mutant U2AF1(S34F)-induced haematopoietic progenitor cell expansion by colony-forming unit (CFU-C) assay (Fig. 3b) and by flow cytometry for lineage-, c-Kit+, Sca1+ (KLS) cells (Fig. 3c) when compared with control U2AF1 mutant mice treated with vehicle and mice transplanted with U2AF1(WT)-expressing bone marrow. The attenuation of mutant U2AF1-induced progenitor expansion by sudemycin-treated mice is associated with increased Annexin V+ staining of KLS cells (Fig. 3d).

### Sudemycin and U2AF1 (S34F) splicing effects can be cumulative

**Figure 4:**
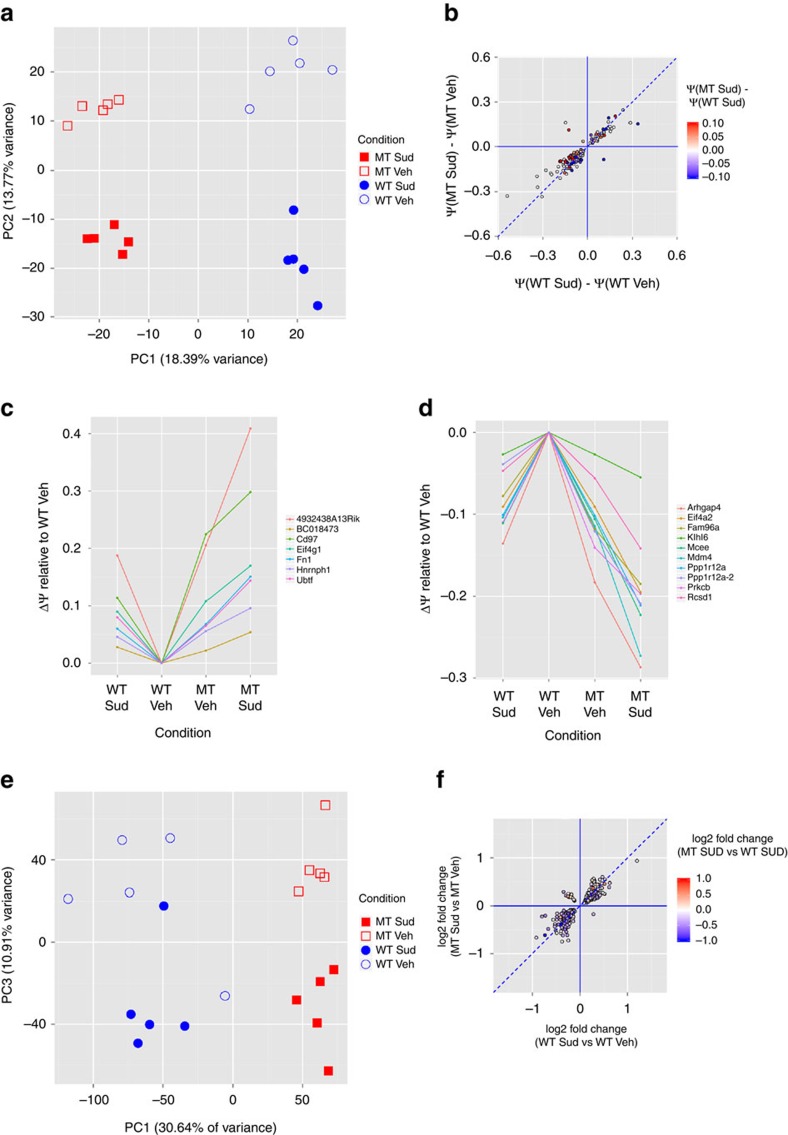
Sudemycin D6 treatment alters splicing and gene expression in mutant U2AF1-haematopoietic cells. RNA sequencing and transcriptome analysis was performed on RNA harvested from mouse bone marrow cells expressing mutant U2AF1(S34F) or U2AF1(WT) following treatment of mice with sudemycin D6 (50 mg kg^−1^) or vehicle control for 5 days (*n*=5 per genotype and treatment). (**a**) PCA of normalized expression of skipped cassette exon events. (**b**) Correlation between sudemycin-induced changes relative to vehicle treatment in cassette exons of U2AF1(WT) cells 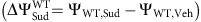 (FDR<10%) and of mutant U2AF1(S34F) cells 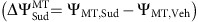 (FDR<10%; *R*^2^=0.8; *P*<2.2 × 10^−16^, F-test). Dashed line indicates similar effects in U2AF1(WT) and U2AF1(S34F) cells 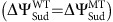. Colour scale indicates cumulative splicing changes of U2AF1(S34F) expression with sudemycin treatment (that is, 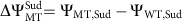), with red being a positive change, blue being a negative change, and white being no difference between sudemycin-treated U2AF1(S34F) and U2AF1(WT) cells. (**c**,**d**) Delta PSI (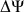) for each condition on the horizontal axis relative to vehicle-treated U2AF1(WT) cells ((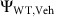)=0) for events that are significantly dysregulated across all five comparisons (see Supplementary Methods), have concordant sudemycin-induced dysregulation across genotype (that is, 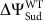 and 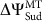 have the same direction), and in which the sudemycin effect is exacerbated by mutation. The cumulative effect on cassette exons induced by both mutant U2AF1 and sudemycin D6 treatment relative to vehicle-treated WT cells, 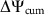, may result in increased exon skipping (positive values, **c**) or increased exon inclusion (negative values, **d**). (**e**) PCA of normalized expression of expressed genes. (**f**) Correlation between sudemycin-induced changes relative to vehicle treatment (expressed as log_2_ fold changes, FDR<10%) in U2AF1(WT) and U2AF1(S34F) cells (*R*^2^=0.8; *P*<2.2 × 10^−16^, F-test). Dashed line indicates log_2_ fold changes induced by sudemycin are the same in U2AF1(WT) and U2AF1(S34F) cells. Colour scale indicates cumulative contribution of mutant U2AF1 expression to sudemycin-induced gene expression changes, that is, log_2_ fold change of gene expression altered by sudemycin in mutant U2AF1(S34F) cells relative to U2AF1(WT) cells, with red being a positive change, blue being a negative change and white being no difference. U2AF1 mutant (MT), sudemycin D6 (Sud), U2AF1 WT, vehicle (Veh), principal component 1 (PC1), principal component 2 (PC2), principal component 3 (PC3), per cent spliced in (PSI, Ψ).

To investigate the potential genotype-specific effects of sudemycin treatment on splice isoform expression, we performed whole-transcriptome sequencing (RNA-seq) on U2AF1(S34F)- and U2AF1(WT)-recipient mouse bulk bone marrow cells following *in vivo U2AF1* transgene induction and treatment with sudemycin D6 (50 mg kg^−1^ per day for 5 days) or vehicle (Supplementary Fig. 3a,b). RNA was harvested 18 h after the last drug treatment (similar to described above; schema shown in Fig. 3a). Sudemycin D6 treatment at this dose and schedule does not markedly skew the mature lineage distribution within bulk bone marrow of mutant or wild-type (WT) U2AF1 transgenic mice (Supplementary Fig. 3c). Using an unsupervised approach, we observed that sudemycin D6 perturbs splicing in both mutant U2AF1(S34F) and U2AF1(WT)-expressing bone marrow cells (Supplementary Data 5–9); this is visualized by the segregation of samples according to genotype and treatment within a principal component analysis (PCA) of cassette exon (Fig. 4a) and retained intron (Supplementary Fig. 4a) splicing events. Furthermore, the splicing bias observed in human cells treated with sudemycin (described above) is recapitulated in U2AF1(WT) mouse cells: sudemycin D6 induces exon skipping more often than exon inclusion relative to vehicle (388 of 657 significant (FDR<10%, |ΔΨ|>1%) events; *P*<2 × 10^−6^, one-sided binomial test), as well as intron retention more often than removal (98 of 145 significant (FDR<10%, |ΔΨ|>1%) events; *P*<1.4 × 10^−5^, one-sided binomial test). As in human CD34+ cells, the sudemycin-induced changes were not associated with an apparent sequence motif (Supplementary Fig. 4b); however, we did observe the previously reported increase in a T in the −3 position of the intronic 3′ splice acceptor site of exons more commonly skipped in mutant U2AF1(S34F) cells^16^,^17^,^18^,^19^ (Supplementary Fig. 4c). In addition, we defined ‘high-confidence' sets of U2AF1(S34F) and sudemycin targets, and subsets of those had a high validation rate in orthogonal experimental (NanoString^26^) and statistical (edgeR^27^) platforms (Supplementary Information and Supplementary Data 10 and 11).

Next, we examined potential interactions between the drug and mutation within cells, focusing on exon skipping events. Along these lines, we observed that the exon skipping effects induced by sudemycin D6 (relative to vehicle) within U2AF1(WT)-expressing cells are highly correlated with the drug effects in U2AF1(S34F)-expressing cells (*R*^2^=0.8, *P*<2.2 × 10^−16^, F-test, events significant in both comparisons (FDR<10%), Fig. 4b). The vast majority of these events are concordant (in the same direction of induced change with similar magnitude) across genotypes (slope of the regression line=0.75), suggesting that sudemycin treatment results in similar splicing alterations in these targets in both mutant U2AF1 and WT cells (Fig. 4b). We further assessed drug/genotype interaction using a statistical linear model: of 32,529 dysregulated splicing events (across all event types), only 136 showed statistically significant evidence of interaction (that is, synergy or antagonism; DEXSeq, FDR<10%). However, when sudemycin D6 and mutant U2AF1(S34F) dysregulate a junction in the same direction (for example, both increasing exon skipping), this results in a cumulative effect in a sudemycin-treated mutant cell that is greater than the effect induced by sudemycin treatment of WT cells (indicated by red and blue colour in Fig. 4b–d). As an example, U2AF1(S34F) expression induces increased exon skipping in a *4932438A13Rik* splice junction compared with U2AF1(WT) in vehicle-treated cells (ΔΨ=0.205, middle two columns in Fig. 4c). Sudemycin D6 also increases exon skipping of the same junction in U2AF1(WT) (ΔΨ=0.188) and U2AF1(S34F) (ΔΨ=0.204) cells (that is, sudemycin-induced exon skipping is independent of genotype; Fig. 4c). Therefore, as expected, the cumulative effect induced by both mutation and drug treatment (ΔΨ=0.409) exceeds the individual effects of mutant U2AF1 expression or sudemycin treatment alone, ultimately resulting in different levels of exon skipping in sudemycin-treated cells expressing mutant U2AF1 versus WT cells (Fig. 4c). The cumulative effect of mutation and drug can also be observed in splicing events that result in increased exon inclusion (Fig. 4d). Together, these data signify that the effects of sudemycin and U2AF1(S34F) on splicing in the same cell can be cumulative—that is, greater than the separate effects of drug or mutant.

### Sudemycin induces gene expression alterations

Sudemycin treatment *in vivo* also results in altered gene expression in mutant U2AF1 mouse bone marrow cells compared with U2AF1(WT)-expressing control cells (Supplementary Data 12). As with splicing alterations, mouse bone marrow samples segregate by genotype and by treatment in an unsupervised analysis of gene expression (Fig. 4e). As seen at the junction level, there is a high-degree of correlation between sudemycin-induced, gene-level effects (direction and magnitude relative to vehicle) across mutant and WT genotypes (*R*^2^=0.8, *P*<2.2 × 10^−6^, F-test, events significant (FDR<10%) in both comparisons, Fig. 4f). We also observed cumulative effects of mutation and drug on gene expression (colour in Fig. 4f), which may result in downstream cellular pathway changes. Using GOseq, we identified pathways that were most enriched for differentially expressed genes in mutant cells treated with sudemycin D6 relative to the other genotype and treatment groups (FDR<10%). We found mutant cells treated with sudemycin D6 were enriched in biologic pathways related to immune and inflammatory responses, antigen processing and presentation, cytokine production, leukocyte differentiation, cell death and apoptotic processes, when compared with the other genotype and treatment groups (Supplementary Data 13).

## Discussion

We provide evidence suggesting that U2AF1(S34F)-expressing cells are sensitive to the splicing modulator drug sudemycin. Haematopoietic cells expressing mutant U2AF1 have reduced survival and cell cycle changes following sudemycin D6 treatment *in vitro*. *In vivo*, sudemycin D6 is capable of attenuating mutant U2AF1-associated expansion of haematopoietic progenitors in transgenic mice that is associated with increased cell death. In addition, while the effects of sudemycin D6 treatment on splicing and gene expression can be independent of the effects of mutant U2AF1 expression, sudemycin D6 treatment can also modulate splicing changes induced by mutant U2AF1(S34F) to create cumulative effects on cells *in vivo*. Together, these data indicate that mutant U2AF1(S34F)-expressing cells may have a therapeutic vulnerability to splicing modulator drugs such as sudemycin.

Previous studies using splicing modulator drugs, as well as non-pharmacological methods to target splicing factors, have indicated that the spliceosome is a promising therapeutic target for many cancer cell types^13^,^14^,^28^,^29^,^30^,^31^. Recent studies have suggested that cancers with spliceosome gene mutations may have an increased sensitivity to splicing modulator drugs like sudemycin;^32^,^33^,^34^ this study provides further evidence to support this hypothesis. Specifically, we show that mutant U2AF1 cells are sensitive to *in vivo* treatment with sudemycin. Using unbiased RNA-seq, we show that sudemycin D6 has effects on mutant U2AF1-associated splicing changes, resulting in different levels of transcript isoforms in cells expressing mutant U2AF1 compared with WT following sudemycin treatment. Ultimately, cumulative changes in isoform expression could be the cause of the mutant U2AF1-specific responses to sudemycin treatment that we observe *in vitro* and *in vivo*. Identification of critical downstream targets of sudemycin *in vivo* will likely require examination of various cellular populations within the bone marrow (including haematopoietic stem and progenitor cells) and harvesting cells at more immediate time points following drug treatment—both limitations of the current study. Future studies will focus on generating mutant U2AF1-expressing leukaemias in mice to test the efficacy of sudemycin on fully transformed haematopoietic tumours, as has recently been reported using E7107 to treat Srsf2(P95H)-expressing leukaemias^33^.

Our data and others highlight several possible mechanisms for the sensitivity of spliceosome mutant samples to splicing modulator therapy. Pathway analysis of differentially expressed genes induced by sudemycin revealed enrichment in inflammatory signalling pathways. This is consistent with the enrichment in biological pathways related to cytokine and immune signalling observed following *in vivo* treatment of Srsf2(P95H) mutant mice with the splicing modulator E7107 (ref. 33), raising the possibility that spliceosome mutant-specific phenotypes observed in mice following splicing modulator drug treatment may be driven by an altered inflammatory response in mutant cells. Whether the altered inflammatory response in mutant cells treated with splicing modulator drugs is a direct result of mutant-altered splicing or gene expression could be explored in future studies. Alternatively, it is also possible that the cumulative effect of sudemycin and mutant U2AF1 expression on pre-mRNA splicing may simply create a state of ‘spliceosome sickness' in cells by exceeding a tolerable threshold of splicing perturbations and their downstream consequences. Along these lines, other splicing modulator drugs have been shown to cause increased intron retention, R loop formation, DNA damage and cell death^28^,^29^,^31^,^35^. Spliceosome gene mutations and splicing modulator drugs may both induce these consequences in a cell, and the cumulative effect of the drug and mutation may create a toxic intracellular milieu.

Exploring the clinical utility of splicing modulator therapies in MDS patients with spliceosome mutations who have failed current therapies is warranted and currently being pursued (NCT02841540), as these patients have few treatment options. Whether excessive toxicity will occur in WT cells treated with newer splicing modulators is a major question. Initial phase I clinical trial studies using a splicing modulator, E7107, showed toxicities in some patients with solid tumours, including ocular toxicity (NCT00459823, NCT00499499). The mechanism for the toxicity is not known. Moving forward, mutant U2AF1 and other preclinical models of spliceosome mutations will be valuable in further testing the *in vivo* efficacy and toxicity of drugs that modulate splicing through various mechanisms. Collectively, our data suggest that a mutant splicing factor together with a splicing modulator drug like sudemycin may create a unique cellular toxicity that could be exploited for therapeutic purposes.

## Methods

### Isolation and drug treatment of CD34+ haematopoietic cells

Mononuclear cells from human umbilical cord blood were separated by Ficoll gradient centrifugation (each n value is a pooled set of four individual umbilical cord blood samples). CD34+ cells were enriched using autoMACS-positive selection (CD34 MicroBead Kit, Miltenyi Biotec) according to the manufacturer's instructions to achieve >90% purity of CD34+ cells. Following isolation, CD34+ cells were cultured in *X-Vivo* media (Lonza) with cytokines (SCF, FLT3L, IL3 and TPO) overnight. For whole-transcriptome sequencing with sudemycin, either sudemycin D6 (1,000 nM) or the vehicle DMSO was added to cells for 6 h, and cells were then harvested for RNA. Tissue acquisition was performed per protocol approved by the Washington University School of Medicine Institutional Review Board.

### RNA-seq of CD34+ cells treated with sudemycin

RNA was isolated from human CD34+ cells using the miRNEasy Kit (Qiagen), and removal of genomic DNA was performed via a Turbo DNA-Free Kit (Ambion). Ribosomal RNA was removed using Ribozero (Epicenter), followed by cDNA preparation and generation of stranded libraries using the TruSeq Stranded Total RNA Sample Prep Kit (Illumina). Sequencing was performed on the HiSeq2500 platform (Illumina) to generate 2 × 125 bp paired-end reads. RNA-seq data were deposited in NCBI dbGAP (phs000159.v9).

### RNA-seq analysis of human CD34+ cells treated with sudemycin

Analysis of (stranded) RNA-seq data generated from primary human CD34+ haematopoietic cells treated with sudemycin D6 was performed using the genome modelling system^36^. Reads were aligned to the human genome (hg19/NCBI build 37) using TopHat^37^ version 2.0.8, with annotations provided by Ensembl^38^ version 67. All downstream bioinformatic and statistical analyses, including calculation of *P* values using Fisher's exact test and simulation, were performed in R^39^ and python. The heatmap of gene expression was created using normalized expression values output by DESeq2 package (version 1.6.3 (ref. 40)) that was subsequently *z*-scored. DESeq2 was used to detect differentially expressed genes, and gene-set enrichment analysis was then performed by applying GOseq^41^ (version 1.18.0) to differentially expressed genes (FDR<5%, |log_2_FC|>1, DESeq2). Additional details of RNA-seq analysis of gene expression are further described in the Supplementary Methods.

Alternative splicing events were computed via rMATS^42^ (version 3.0.8). We generated the heatmap of splice junctions using ‘per cent spliced in' (PSI or Ψ) values for exon skipping and intron retention events. For downstream analyses, namely reported dysregulated events and visualization of sequence contexts below, *P* values for events passing the filter were re-adjusted for multiple hypothesis testing using the method of Benjamini and Hochberg^43^. A positive ΔPSI value indicates an event that was spliced in more often in the vehicle-treated relative to the sudemycin-treated samples. Sequence contexts of splice sites in exon skipping and intron retention events were visualized using seqLogo version 1.32.1. Dysregulated events visualized were those with |ΔPSI|>10% and having a post-filtering re-adjusted FDR<5% from rMATS. Dysregulated splicing junctions were determined by DEXSeq^4^ version 1.12.2. Only expressed junctions were analysed, and gene-set enrichment analysis was performed by applying GOseq to dysregulated junctions (FDR<5%, |log_2_FC|>1, DEXSeq). All of these are described in more detail in the Supplementary Methods.

### Creation of inducible U2AF1(S34F) and U2AF1(WT) cell lines

Doxycycline-inducible FLAG-tagged WT U2AF1 or FLAG-tagged mutant U2AF1(S34F) lentiviral expression plasmids were previously described^4^, and lentivirus was generated in 293T cells with the packaging plasmids pMD-G, pMD-Lg and REV. Concentrated virus (multiplicity of infection (MOI) of 3 for K562, MOI of 5 for OCI-AML3) was used to transduce K562 cells (ATCC, CCL243) or OCI-AML3 (DSMZ, ACC 582). Transduced cells (marked by green fluorescent protein) were isolated by flow cytometry cell sorting. Expression of mutant or WT U2AF1 was induced using the indicated concentrations of doxycycline hyclate (Sigma, St Louis, MO) in water.

### Cell line counting and BrdU incorporation assays

K562 (or OCI-AML3) cells in culture were seeded at ∼200,000 cells per ml with different concentrations of Sudemycin D6 (or with DMSO control) following 48 h of initial doxycycline treatment to induce mutant U2AF1(S34F) or U2AF1(WT) expression. Cells were counted on Days 0, 3 and 5 of drug treatment using flow cytometry counting particles (Spherotech) as per the manufacturer's recommendations, along with propidium iodide (Millipore) to exclude dead cells from counts. Day 5 data were graphed in Graphpad Prism (Graphpad Software Inc.) using a nonlinear regression best fit line of the log (drug) versus response for each genotype and treatment. Statistical differences between curves were examined by the extra sum-of-squares F-test function in Graphpad Prism. For K562 cells, 5-bromodeoxyuridine (BrdU) incorporation was performed using the BrdU Flow Kit (APC, #559619, BD Biosciences) as per the manufacturer's instructions using a 45 min pulse of BrdU of cells in culture on Day 5 of drug treatment.

### Culture of MDS/AML cells and EdU incorporation assay

Primary MDS and AML cells were cultured on irradiated (4,000 cGy) HS27 stroma as described previously^45^. Briefly, MDS or AML cells were cultured on stroma for 2–3 days and then treated with increasing doses of sudemycin D1 (or daunorubicin in Supplementary Information). MDS and AML cells were cultured with drug for 3 days and analysed for cell proliferation by EdU incorporation assay (Invitrogen) via flow cytometry. All experiments were performed on 96-well plates. Studies were performed per protocol approved by the Washington University School of Medicine Institutional Review Board and with patients' consent for sample use.

### Murine bone marrow transplant and drug infusion

To generate mice for each experiment, 1 × 10^6^ transgenic mouse donor bone marrow cells from two to three mice pooled (CD45.2) were transplanted into at least five lethally irradiated (1,100 rads) congenic WT recipient mice (C57BL/6 × 129S4Sv/Jae)F1 (CD45.1/CD45.2) per genotype, as previously described^19^. Donor mice were between 8 and 12 weeks of age, and recipient mice ranged from 6 to 12 weeks of age; donor and recipient mice were sex-matched (both sexes were used in experiments). Donor chimerism was confirmed ≥6 weeks post transplantation to ensure engraftment of transgenic bone marrow. Post-engraftment, intrajugular catheters were surgically placed for intravenous drug infusions of mice over 4 h daily for 5 days with either sudemycin D6 (50 mg kg^−1^) or vehicle control (HP-β-CD (2-hydroxypropyl)-β-cyclodextrin in phosphate buffer pH 7.4). This dose was determined by prior studies^13^. Mice received doxycycline chow to induce U2AF1(S34F) or U2AF1(WT) transgene on the day of surgery, and drug infusion began 2 days later. Mice of each group were randomly selected for treatment with sudemycin or vehicle control. Investigators were not blinded to the group allocation during the experiment or analysis. Animals were excluded from analysis if their catheters did not remain patent during the entire treatment period. Mice were euthanized for analysis the day following the last drug infusion. Sample sizes for experiments were chosen to allow for statistical comparison between groups. All mouse procedures were performed according to the protocols approved by the Washington University Animal Studies Committee.

### Mouse haematopoietic progenitor cell assay

Methylcellulose progenitor CFU-C assays were performed using Methocult GF M3434 (Stem Cell Technologies). Bulk bone marrow cells were obtained from experimental mice, and red blood cells were lysed before plating of 10,000 bone marrow cells per 1.3 ml media; each sample was evaluated in duplicate. Progenitor colonies (defined as ≥40 cells per colony) were counted following 7 days culture at 37 °C with 5% CO_2_.

### Mouse haematopoietic cell flow cytometry

For flow cytometry, all antibodies are from eBioscience (unless indicated), and catalogue number provided (if available). For haematopoietic progenitor/stem cells, we used the following antibodies (volume of antibody used in 200 μl of fluorescence-activated cell sorting (FACS) buffer for staining is also indicated): CD45.1-APC (#17-0453, 3 μl), CD45.2-PE (#12-0454, 3 μl), Biotin-conjugated lineage (Gr-1 (#13-5931, 0.25 μl), Cd3e (#13-0032, 0.5 μl), B220 (#13-0452, 0.5 μl), Ter119 (#13-5921, 0.5 μl) and CD41(#13-0411, 1 μl)), streptavidin secondary-eFluor605NC, c-Kit-APCeFluor780 (#47-1172, 1.5 μl), Sca1-PerCP-Cy5.5 (#45-5981, 0.5 μl). Flow cytometry for Annexin V+ staining of KLS cells was performed following initial staining of KLS cells as described above using Annexin V-APC (2.5 μl, BD Biosciences, #550474) incubated in 1 × Annexin V binding buffer (BD Biosciences). All other incubations occurred in FACS buffer. All flow cytometry analyses were performed using FACScan or Gallios cytometers (BD Biosciences) and analysed using the FlowJo software (FlowJo, LLC, Ashland, OR, USA).

### RNA-seq of mouse bone marrow cells *in vivo*

RNA was isolated and prepared as described above for human cells, using the miRNEasy Kit (Qiagen) followed by removal of genomic DNA via a Turbo DNA-Free Kit (Ambion) and ribosomal RNA using Ribozero (Epicenter). The cDNA preparation and generation of stranded libraries was performed using the TruSeq Stranded Total RNA Sample Prep Kit (Illumina). Sequencing was performed on the HiSeq2500 platform (Illumina) to generate 2 × 126 bp paired-end reads. The RNA-seq data have been deposited in NCBI's Gene Expression Omnibus^46^ and are accessible through the GEO Series accession number GSE89834.

### RNA-seq analysis of mouse bone marrow cells *in vivo*

Analysis of (stranded) RNA-seq data generated from U2AF1(S34F) and U2AF1(WT) murine cells treated with sudemycin D6 or vehicle was performed similarly to the human CD34+ cell analysis described above, with some differences as follows. Alignment again utilized genome model system, and reads were aligned to the mouse genome (mm9/NCBI build 37). ‘Per cent spliced in' (PSI or 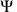) values were calculated for all four conditions ({U2AF1(S34F), U2AF1(WT)} × {sudemycin D6, vehicle}) using rMATS. PCA was performed independently on events annotated by rMATS as skipping cassette exons or retaining introns using the *z*-scored Ψ values of these events.

To quantitate the simultaneous effect of treatment and genotype for each splicing event, we calculated the change in per cent spliced in values for the four pairwise comparisons in which the genotype (alternately, treatment) was the same in the pair, but in which the treatment (alternately, genotype) differed. We refer to the unchanged condition as the ‘context' and to the two conditions that differ as ‘A' and ‘B' and denote the corresponding change in the per cent spliced in values as 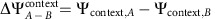. In addition, we defined the cumulative effect in a mutant, drug-treated cell relative to a WT, vehicle-treated cell as 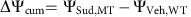. Each of the five comparisons described were evaluated using rMATS, and *P* values for these events were then adjusted for multiple hypothesis testing as described above. Scatterplots were then plotted of cassette exon-skipping events dysregulated by sudemycin in both U2AF1(WT) and U2AF1(S34F) contexts (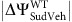
>1%, 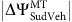
>1%, FDR<10% in both comparisons). To highlight cumulative cassette exon-skipping effects, ‘trajectories' comparing Ψ values to the ‘baseline' 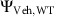 were plotted. Events that were significant in all five comparisons (that is, 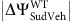
>1%, 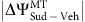
>1%, 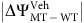
>1%, 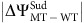
>1%, 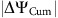
>1%, with FDR<10% in all comparisons) were plotted; the latter condition ensures that visually discernible differences are statically significant and not attributable to statistical noise. Additional details of this can be found in the Supplementary Methods.

Non-additive (that is, super-additive synergistic or sub-additive antagonistic) interactions between drug and mutation were assessed in DEXSeq by comparing a generalized linear model that included main effects for drug and mutation with a second model that additionally included an interaction term representing drug/mutation synergy or antagonism.

As above, splice site sequence contexts of exon-skipping events dysregulated by sudemycin D6 (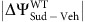
>1%, FDR<10%) and corresponding unperturbed control events (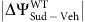
>0.1%, FDR>50%) were plotted using seqLogo. Similarly, sequence contexts were displayed for events dysregulated (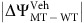
>1%, FDR<10%) or unaffected (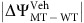
>1%, FDR>50%) by U2AF1(S34F).

At the gene-level, PCA was performed as described above for human expressed genes. Genes differentially expressed within the above-described five conditions were determined via DESeq2. Scatterplots were then made comparing log_2_ fold changes of genes dysregulated by sudemycin in both U2AF1(WT) and U2AF1(S34F) contexts (FDR<10% in both comparisons). Pathways enriched (FDR<10%) for genes differentially expressed between U2AF1(S34F), sudemycin-treated cells and U2AF1(WT) and/or vehicle-treated cells were independently determined in a pairwise manner using GOseq. Again, expanded details of this approach can be found in the Supplementary Methods.

### Data availability

All relevant data generated in this study are available at data-deposition sites. For human CD34+ cells treated with sudemycin D6 *in vitro*, data are available at NCBI dbGAP (phs000159.v9). For transgenic mice expressing mutant U2AF1(S34F) or U2AF1(WT) and treated with sudemycin D6 *in vivo*, data are available at Gene Expression Omnibus (GSE89834).

## Additional information

**How to cite this article:** Shirai, C. L. *et al*. Mutant U2AF1-expressing cells are sensitive to pharmacological modulation of the spliceosome. *Nat. Commun.* **8,** 14060 doi: 10.1038/ncomms14060 (2017).

**Publisher's note**: Springer Nature remains neutral with regard to jurisdictional claims in published maps and institutional affiliations.

## Supplementary Material

Supplementary Information Supplementary Figures, Supplementary Methods and Supplementary References

Supplementary Data 1 Genes differentially expressed (DESeq, FDR<5%, |log2FC>1|) in primary human CD34+ hematopoietic cells treated with Sudemycin D6.

Supplementary Data 2 Differentially spliced junctions (FDR<5%, |delta percent spliced in or PSI (Δψ)|>10%) in primary human CD34+ hematopoietic cells treated with Sudemycin D6.

Supplementary Data 3 Pathways enriched (GOseq FDR < 0.1) in differentially spliced genes in primary human CD34+ hematopoietic cells following treatment with sudemycin D6.

Supplementary Data 4 Pathways enriched (GOseq FDR < 0.1) in differentially expressed genes in primary human CD34+ hematopoietic cells following treatment with sudemycin D6.

Supplementary Data 5 Differentially spliced junctions (FDR<10%, |delta percent spliced in or PSI (Δψ)|>1%) between vehicle-treated mutant and wildtype U2AF1-expressing bone marrow cells in vivo.

Supplementary Data 6 Differentially spliced junctions (FDR<10%, |delta percent spliced in or PSI (Δψ)|>1%) between vehicle-treated and sudemycin D6-treated wildtype U2AF1-expressing bone marrow cells in vivo.

Supplementary Data 7 Differentially spliced junctions (FDR<10%, |delta percent spliced in or PSI (Δψ)|>1%) between vehicle-treated and sudemycin D6-treated mutant U2AF1-expressing bone marrow cells in vivo.

Supplementary Data 8 Differentially spliced junctions (FDR<10%, |delta percent spliced in or PSI (Δψ)|>1%) between sudemycin-treated mutant and wildtype U2AF1-expressing bone marrow cells in vivo.

Supplementary Data 9 Differentially spliced junctions (FDR<10%, |delta percent spliced in or PSI (Δψ)|>1%) between vehicle-treated wildtype U2AF1- and sudemycin D6-treated mutant U2AF1-expressing bone marrow cells in vivo.

Supplementary Data 10 List of "high confidence" splice junctions that are differentially spliced by mutant U2AF1(S34F) in mouse bone marrow cells.

Supplementary Data 11 List of "high confidence" splice junctions that are differentially spliced by sudemycin D6 in mouse bone marrow cells.

Supplementary Data 12 Genes differentially expressed between vehicle-treated and drug-treated wildtype U2AF1- and mutant U2AF1-expressing bone marrow cells (FDR<10%) in vivo.

Supplementary Data 13 Pathways enriched (GOseq FDR < 0.1) in genes differentially expressed between vehicle-treated and drug-treated wildtype U2AF1- and mutant U2AF1-expressing bone marrow cells in vivo.
