# Supplementary material for: Mutant U2AF1-expressing cells are sensitive to pharmacological modulation of the spliceosome
Source: Nat Commun. 2017 Jan 9;8:14060. doi: 10.1038/ncomms14060 (PMC5227701; doi:10.1038/ncomms14060)
Supplement: Supplementary Information — Supplementary Figures, Supplementary Methods and Supplementary References [file ncomms14060-s1.pdf]

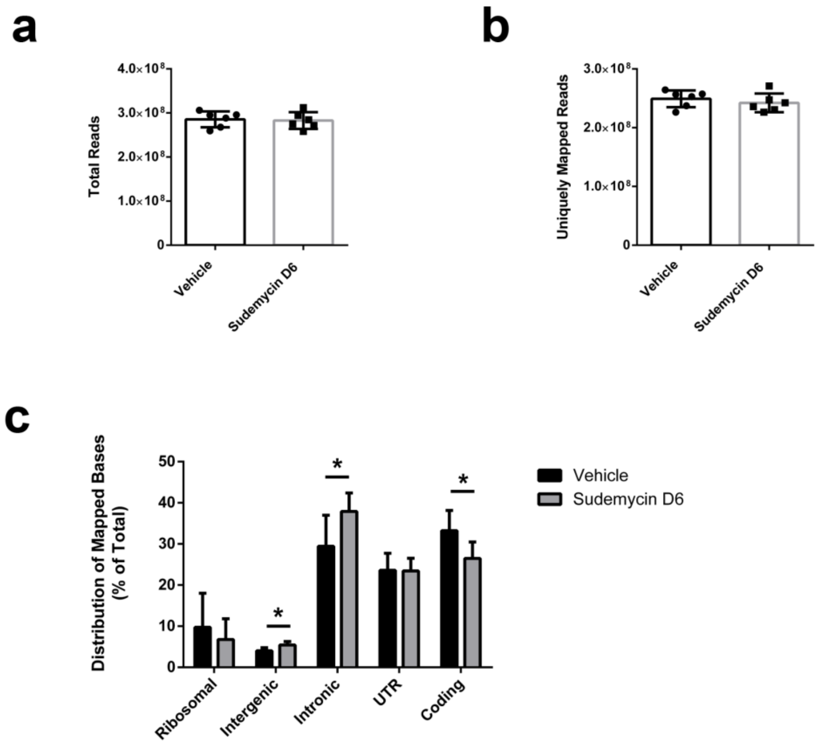

Supplementary Figure 1. RNA sequencing metrics of primary human CD34+ hematopoietic cells treated with sudemycin D6 or drug vehicle (a) Total reads and (b) uniquely mapped reads from RNA-seq samples (n=6 pools of 4 umbilical cords each) treated for 6 hours with sudemycin D6 (1000 nM) or vehicle control (DMSO). (c) Distribution of mapped bases from CD34+ RNA-seq samples. UTR = untranslated region, * $p<0.05$, statistics calculated using two-tailed t-tests for each comparison shown; mean values with s.d. shown.

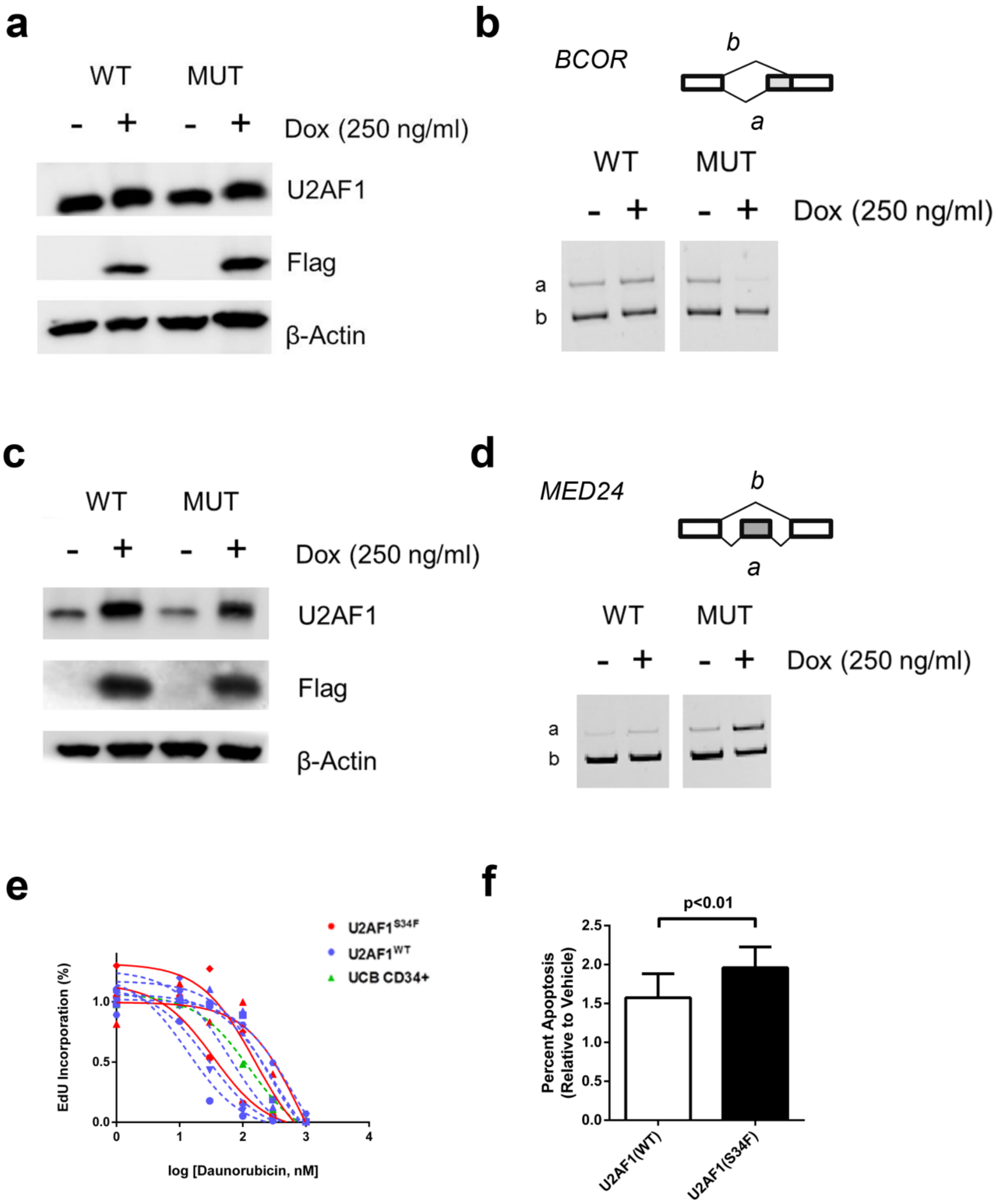
a
WT
MUT
- + - +
Dox (250 ng/ml)
U2AF1
Flag
β-Actin
b
BCOR
b
a
WT
MUT
- + - +
Dox (250 ng/ml)
a
b
c
WT
MUT
- + - +
Dox (250 ng/ml)
U2AF1
Flag
β-Actin
d
MED24
b
a
WT
MUT
- + - +
Dox (250 ng/ml)
a
b
e
U2AF1 S34F
U2AF1 WT
UCB CD34+
EdU Incorporation (%)
log [Daunorubicin, nM]
f
p<0.01
Percent Apoptosis (Relative to Vehicle)
U2AF1(WT)
U2AF1(S34F)

Supplementary Figure 2. The sensitivity of mutant U2AF1(S34F)-expressing cells to splicing modulator drugs in vitro is mutant-specific For induction of either U2AF1(WT) or U2AF1(S34F) in K562 and OCI-AML3 cells, cultures were treated for 48 hours with 250 ng/ml of doxycycline (Dox). (a, c) Protein lysates from K562 and OCI-AML3 cells were assayed by Western blot for U2AF1 and FLAG epitopes, as well as β-actin for protein loading control, respectively. (b, d) RNA from treated K562 or OCI-AML3 cells was analyzed by RT-PCR and gel electrophoresis for mutant U2AF1-associated splicing changes in known target genes: (b) *BCOR* was examined in K562 cells, (d) *MED24* was examined in OCI-AML3 cells. A schematic of each splicing event is also shown. (e) Primary human MDS or AML cells [both mutant U2AF1(S34F) samples (n=3) and those wildtype for U2AF1 (n=6)] or normal umbilical cord blood CD34+ cells (n=1) were cultured on irradiated HS27 stroma and proliferation (EdU incorporation) was measured after 3 days of exposure to increasing concentrations of daunorubicin. (f) CD34+ hematopoietic cells isolated from human umbilical cord blood samples were transduced with either U2AF1(WT) or U2AF1(S34F)-expressing lentivirus and treated with the splicing modulator drug E7107 (15 nM) for 24 hrs. Apoptosis was analyzed by flow cytometry for Annexin V+/7-AAD (n=9). Statistics calculated using two-tailed t-test; mean values with s.d. shown.

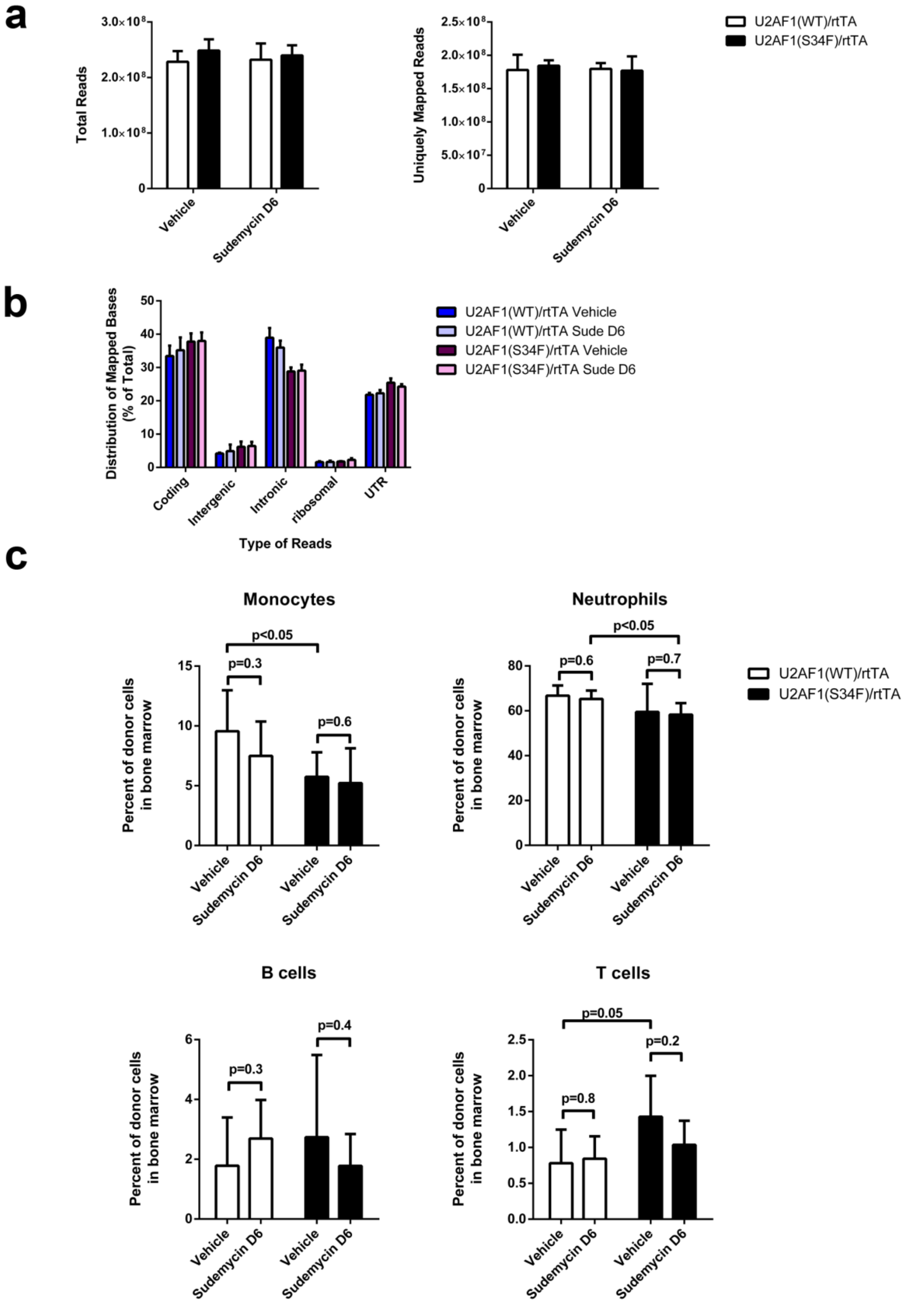
a
Total Reads
3.0×10^8
2.0×10^8
1.0×10^8
0
Vehicle
Sudemycin D6
Uniquely Mapped Reads
2.5×10^8
2.0×10^8
1.5×10^8
1.0×10^8
5.0×10^7
0
Vehicle
Sudemycin D6
U2AF1(WT)/rtTA
U2AF1(S34F)/rtTA
b
Distribution of Mapped Bases (% of Total)
50
40
30
20
10
0
Coding
Intergenic
Intronic
ribosomal
UTR
Type of Reads
U2AF1(WT)/rtTA Vehicle
U2AF1(WT)/rtTA Sude D6
U2AF1(S34F)/rtTA Vehicle
U2AF1(S34F)/rtTA Sude D6
c
Monocytes
Percent of donor cells in bone marrow
p<0.05
p=0.3
p=0.6
15
10
5
0
Vehicle
Sudemycin D6
Vehicle
Sudemycin D6
Neutrophils
Percent of donor cells in bone marrow
p<0.05
p=0.6
p=0.7
80
60
40
20
0
Vehicle
Sudemycin D6
Vehicle
Sudemycin D6
U2AF1(WT)/rtTA
U2AF1(S34F)/rtTA
B cells
Percent of donor cells in bone marrow
p=0.3
p=0.4
6
4
2
0
Vehicle
Sudemycin D6
Vehicle
Sudemycin D6
T cells
Percent of donor cells in bone marrow
p=0.05
p=0.2
p=0.8
2.5
2.0
1.5
1.0
0.5
0.0
Vehicle
Sudemycin D6
Vehicle
Sudemycin D6

Supplementary Figure 3. RNA sequencing metrics and mature cell distribution of mouse bone marrow following in vivo U2AF1(S34F) transgene induction and sudemycin treatment Mice transplanted with doxycycline-inducible U2AF1(S34F) or U2AF1(WT) transgenic mouse bone marrow were treated with sudemycin D6 (50mg/kg for 5 days) or vehicle control concurrently with transgene induction that had started 2 days prior (see schema in Fig 3a). Bulk bone marrow cells were harvested for RNA-seq (n=5 mice per genotype and treatment). (a) Total reads (left panel) and uniquely mapped reads (right panel) from mouse RNA-seq samples, rtTA = reverse tetracycline-controlled trans-activator. (b) Distribution of mapped bases from mouse RNA-seq samples, UTR = untranslated region. (c) Mature hematopoietic cell lineage distribution, as measured by flow cytometry for monocytes (Cd115+, $Gr1^{low}$), neutrophils (Gr1+), B cells (B220+), and T cells (Cd3e+). * $p<0.05$, statistics calculated using two-tailed t-tests for each comparison indicated; mean values with s.d. shown; n=6-11.

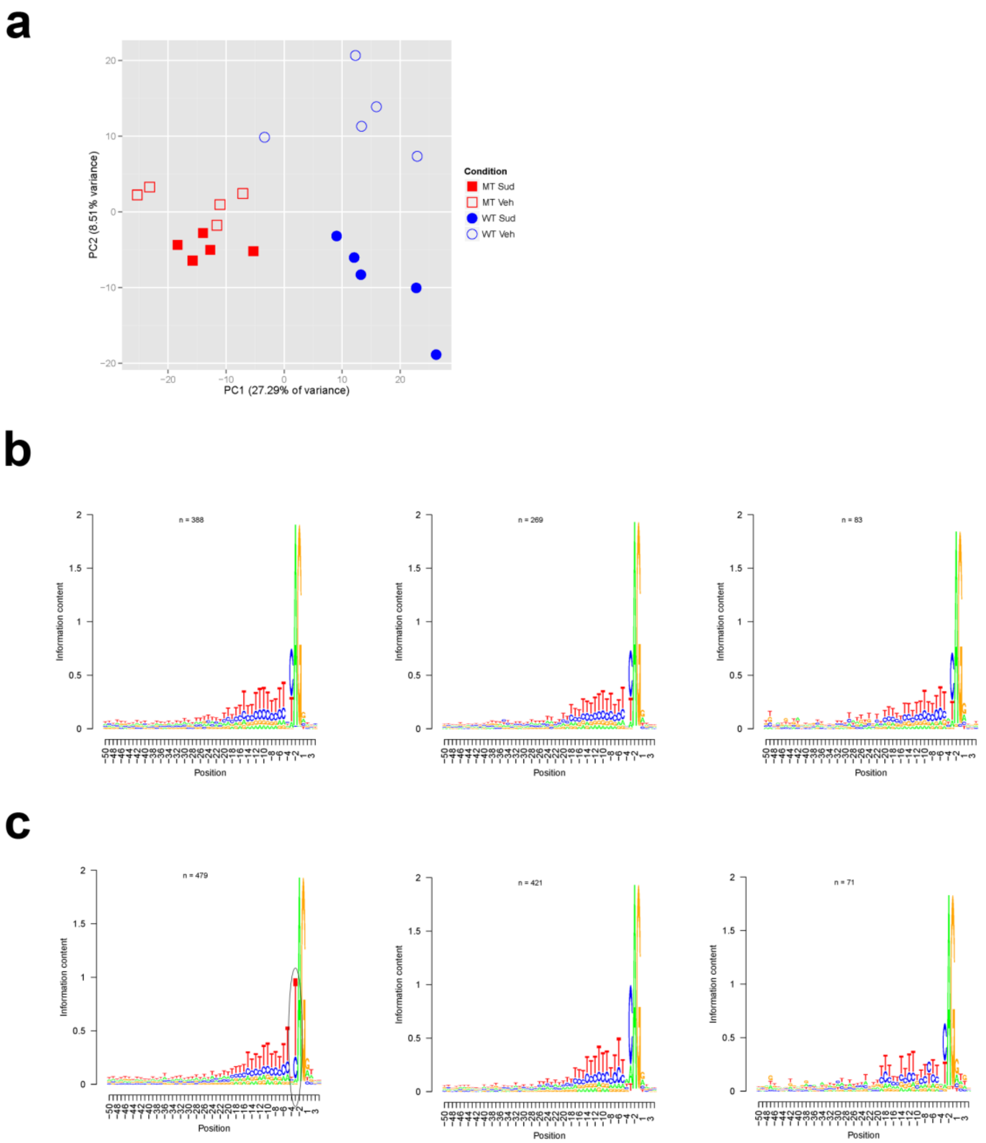

Supplementary Figure 4. Sudemycin D6 treatment alters splicing in mutant U2AF1-hematopoietic mouse cells without recognizing a sequence-specific motif RNA sequencing and analysis was performed on RNA harvested from mouse bone marrow cells expressing mutant U2AF1(S34F) or U2AF1(WT) following treatment of mice with sudemycin D6 (50mg/kg) or vehicle control (n=5 per genotype and treatment). (a) Principal component

analysis (PCA) of normalized expression of retained intron events in mouse samples. MT= mutant U2AF1(S34F)-expressing samples, WT= U2AF1(WT)-expressing samples; Sud= sudemycin D6-treated, Veh= vehicle-treated. (b) Sequence contexts of cassette exon 3' splice sites skipped more often in sudemycin- relative to vehicle-treated cells (FDR<10%, |delta percent spliced in (ΔΨ)|>1%, left panel) or skipped more often in vehicle-treated cells (FDR<10%, |ΔΨ|>1%, middle panel), along with a contexts of unperturbed control exons (FDR>50%, |ΔΨ|<0.1%, right panel). Position is relative to the first base in the exon. (c) Sequence contexts of cassette exon 3' splice sites skipped more often in mutant U2AF1(S34F) samples relative to WT U2AF1 cells (FDR<10%, |ΔΨ|>1%, left panel; circled base indicates the enrichment of exons containing a "T" at the -1 position that tend to be skipped when mutant U2AF1 is expressed) or skipped more often in WT U2AF1 cells compared to U2AF1(S34F) cells (FDR<10%, |ΔΨ|>1%, middle panel), and the contexts of unperturbed control exons (FDR>50%, |ΔΨ|<0.1%, right panel). Again, position is relative to the first base in the exon.

Supplementary Methods

Immunoblotting and pre-mRNA splicing activity assays for cell lines

Soluble whole cell protein lysate was prepared from K562 (or OCI-AML3) cells using lysis buffer (50 nM HEPES, pH 7.5, 150 mM NaCl, 1 mM EDTA, 10% Glycerol, 0.1% Tween-20 plus protease inhibitors) and sonication, and 50 µg of total protein per sample was resolved via SDS-PAGE electrophoresis. Protein was transferred to PVDF membrane (Millipore), and immunoblotting with anti-U2AF1 (Abcam, #ab86305, 1:2000), anti-FLAG (Sigma, #F1804, 1:5000), and anti-β-Actin (Sigma, #AC-15, 1:10000) antibodies was performed per the manufacturer's recommendation. Primary antibody detection was performed using horseradish peroxidase-conjugated secondary antibodies (Cell Signaling, #7074 or #7076, 1:10000). Immunoreactive bands were detected via SuperSignal West Pico (or Femto) Chemiluminescent substrate (ThermoScientific) and imaged using myECL Imager (ThermoScientific).

RNA was isolated from K562 (or OCI-AML3) cells using the miRNEasy kit (Qiagen). Removal of genomic DNA was performed via a Turbo DNA-Free kit (Ambion), and cDNA was prepared using the Superscript III kit (Invitrogen). PCR to detect BCOR or MED24 isoforms affected by U2AF1(S34F) was performed with JumpStart Taq ReadyMix (Sigma-Aldrich) using primers: For BCOR (For 5'-GACAGCAGCCACACTGAGAC-3'; Rev 5'-TCTTCCGACCAGCTTCTGTT-3'; product sizes 296bp, 398bp); For MED24 (For 5'-CACGGCAAAGCAGAGGAATG-3'; Rev 5'-AATGCTCGATGGCAGTCCAA-3'; product sizes 253bp, 310bp). Products were resolved by TBE-polyacrylamide gel electrophoresis.

Culture of CD34+ hematopoietic progenitor cells with E7107 and apoptosis assay

As described above, mononuclear cells from human umbilical cord blood were separated by Ficoll gradient centrifugation. CD34+ cells were enriched using autoMACS

positive-selection (CD34 MicroBead kit, Miltenyi Biotec) according to the manufacturer's instructions to achieve >90% purity of CD34+ cells. Following isolation, CD34+ cells were cultured in media with cytokines (SCF, IL3, Flt3L, TPO all at 50 ng/mL, PeproTech) overnight, and cells were transduced the next morning with U2AF1(WT) or U2AF1(S34F) expressing HIV-lentivirus-IRES-GFP. Following overnight expansion, cells were plated at 50,000 per well on 96 well plates with or without 15 nM E7107 (H3 Biomedicine). Apoptosis evaluated approximately 24 hours following drug treatment by flow cytometry for Annexin V+ (FITC, BD Bioscience) and 7AAD (BD Bioscience), both 7AAD+ and 7AAD- events were plotted.

Mouse mature hematopoietic cell flow cytometry

For flow cytometry, all antibodies are from eBioscience, unless indicated and clone ID provided (if available). For mature lineage cell distribution analysis by flow cytometry, we used the following antibodies from eBioscience, unless otherwise indicated: CD45.1-eFluor450 (#48-0453, 2 µl), CD45.2-FITC (#553772, 0.5 µl, BD Biosciences), CD115-PE (#12-1152, 1 µl), Gr-1-APCeFluor780 (#47-5931, 0.25 µl), B220-PerCPCy5.5 (#45-0452, 0.5 µl), and CD3e-APC (#17-0031, 1 µl). All incubations occurred in 100 µl of FACS buffer. All flow cytometry was performed using FACScan or Gallios cytometers (BD Biosciences) and analyzed using FlowJo software (FlowJo, LLC, Ashland, OR).

Extended methods for RNA-seq analysis of human CD34+ hematopoietic cells treated with sudemycin D6

Analysis of (stranded) RNA-seq data generated from primary human CD34+ hematopoietic cells treated with sudemycin D6 was performed using the genome modeling system.[1] Read trimming, TopHat alignment, and quality checks were performed as listed in processing profile 'a8d8e2cef8e04bf69a58b6333e8c545e' and with results stored in model group '80eca68da5bd484e8a5d9bc0284c952d.' Reads were trimmed by passing parameters '--

adapters adapters.fa --adapter-trim-end RIGHT --length-dist --threads 12 --adapter-min-overlap 7 --max-uncalled 250 --min-read-length 25 --nolength-dist' to FLEXBAR version 2x4, where the file adapters.fa listed the sequences 'AGATCGGAAGAGCGTCGTGTAGGGAAAGAGTGT' and 'AGATCGGAAGAGCACACGTCTGAACTCCAGTCAC'. Reads were then were aligned to the human genome (hg19/NCBI build 37) using TopHat[2] version 2.0.8, with annotations provided by Ensembl[3] version 67. TopHat was passed parameters '--library-type fr-firststrand,' to accommodate stranded reads. All downstream bioinformatic and statistical analyses, including calculation of *p*-values using Fisher's exact test and simulation, were performed in R[4] and python.

Gene counts were determined by excluding secondary read alignments from the BAM files (using samtools view –F 0x0100) and processing the resulting reads using HTseq[5] version 0.5.4p1 (with parameters: mode intersection-strict; minaqual 1; stranded reverse; type exon; idattr gene_id). Heatmap clustering, differential gene expression analysis, and pathway analysis were restricted to expressed genes, defined as those with more than 20 counts in at least half the samples (i.e., six). The heatmap of gene expression displays regularized logarithm values, as output by rLogTransform from the DESeq2 package (version 1.6.3[6]), that were subsequently z-scored. Regularized logarithm values represent count data on a log2 scale that has been normalized for library size and to minimize differences between rows (i.e., genes) having small counts. The heatmap was generated using the pheatmap function of the pheatmap package in R using the UPGMA clustering method (parameter clustering_method = "average") and Pearson's correlation for both the row and column distance metrics (parameters clustering_distance_rows = "correlation" and clustering_distance_cols = "correlation"). Data used in the heatmap were not adjusted to account for the paired experimental design, since the treatment effect clearly dominated any potential batch effect due to the sample pairing. DESeq2 was used to detect differentially expressed genes after introducing a "treatment" term in the

linear model that did, however, account for the paired design. Gene-set enrichment analysis was then performed by applying GOseq[7] (version 1.18.0) to differentially expressed genes (FDR<5%, $|\log_2 FC|>1$, DESeq2).

At the level of splicing, a heatmap was created as above, but using “percent spliced in” (PSI or Ψ) values for exon skipping and intron retention events computed via rMATS[8] (version 3.0.8). rMATS effectively compares two isoforms—an inclusion isoform that splices in a section of mRNA (e.g., an exon or intron) and a skipping isoform that does not. Events included in the heatmap were filtered to ensure that both isoforms were expressed—namely, that each isoform was supported by at least 10 sequencing reads in at least half of the samples (i.e., six samples). This was applied as a post-filtering step of rMATS output, by ensuring that at least half of the inclusion-isoform counts concatenated across the two conditions (vehicle-treated “sample 1” in column IJC_SAMPLE_1 and sudemycin-treated “sample 2” in column IJC_SAMPLE_2) were at least 10. The same filtering was applied independently to skipping-isoform counts in columns SJC_SAMPLE_1 and SJC_SAMPLE_2. Related approaches that require that evaluated isoforms be expressed at some minimal level have been used previously to reduce noise (inherent in low-count data) and to subsequently reduce false positives.[9] For downstream analyses, namely reported dysregulated events and visualization of sequence contexts below, *p*-values for events passing the filter were re-adjusted for multiple hypothesis testing using the method of Benjamini and Hochberg[10] implemented in the R function p.adjust. Throughout, events were those inferred by rMATS using junction-spanning reads only (JunctionCountOnly output files), as opposed those inferred from both junction reads and reads within the alternatively-included mRNA (ReadsOnTargetAndJunctionCount output files). A positive ∆PSI value indicates an event that was spliced in more often in the vehicle-treated relative to the sudemycin-treated samples [i.e., the IncLevel1 column output by rMATS corresponds to the PSI of the vehicle-treated samples, the IncLevel2 column corresponds to the PSI of the sudemycin-

treated samples, and the IncLevelDifference column corresponds to the ΔPSI, or IncLevel1 – IncLevel2].

Splice site sequence contexts of exon skipping and intron retention events were visualized using seqLogo version 1.32.1. Dysregulated events visualized were those with |ΔPSI|>10% and having a post-filtering re-adjusted (above) FDR<5%. rMATS outputs a *p*-value (and an FDR value that adjusts the *p*-value for multiple hypothesis testing) that is the probability that observed ΔPSI value is consistent with the maximum ΔPSI of the null hypothesis, which must be specified for each run of rMATS (via the "c" parameter). i.e., dysregulated events were inferred by passing the parameter "-c 0.1" to rMATS. Control events were those that had an FDR>50% as output from a run of rMATS configured with "-c 0.001"—i.e., these events are unlikely to have a ΔPSI larger than 0.1%.

Dysregulated splicing junctions were determined independent of canonical splicing event type (i.e., cassette exon skipping, mutually exclusive exon usage, intron retention, and alternative 3' or 5' splice site usage) with DEXSeq[11] version 1.12.2. Exon-exon junction counts were obtained by parsing the 'junctions.bed' file produced by TopHat. Only expressed junctions were analyzed [i.e., those with more than ten reads in at least as many samples as in the smallest condition group (here, n=6)]. Gene-set enrichment analysis was then performed by applying GOseq to dysregulated junctions (FDR<5%, $|\log_2 FC|>1$, DEXSeq). As described previously,[12] we attempted to correct for several potential biases that may be correlated with GOseq inference of pathway enrichment: the sum over tested junctions within a gene of the geometric mean of the respective junction's counts[13], the sum over tested junctions within a gene of the arithmetic mean of the respective junction's counts, and the number of tested junctions within a gene. We found the best correlation between the bias and dysregulation ratio using the number of tested junctions bias for the U2AF1(S34F) data, as reported previously.[12]

Extended methods of RNA-seq analysis of transgenic mouse cells treated with sudemycin D6 in vivo

Analysis of (stranded) RNA-seq data generated from U2AF1(S34F) and U2AF1(WT) murine cells treated with sudemycin D6 or vehicle was performed similarly to the above, with differences as follows. Alignment again utilized genome model system processing profile 'a8d8e2cef8e04bf69a58b6333e8c545e.' Here, results were stored in model group '07ed695c708f43999e7a4a67459de9ab.' Trimmed reads were aligned to the mouse genome (mm9/NCBI build 37).

"Percent spliced in" (PSI or Ψ) values were calculated for all four conditions [{ U2AF1(S34F), U2AF1(WT) } x { sudemycin, vehicle }] using rMATS. Principal component analysis (PCA) was performed independently on events annotated by rMATS as skipping cassette exons (SE) or retaining introns (RI). Events were filtered as above to ensure that both isoforms (inclusion and exclusion) were expressed at the level of at least 10 sequencing reads in at least as many samples as involved in each of the four conditions (i.e., five samples across the 20 samples). PCA was performed on the z-scored Ψ values of these filtered events. Specifically, the singular value decomposition of the matrix X, whose columns were z-scored Ψ values, was calculated via svd in R, to yield $X = U S V^T$. The principal components were then computed as U S and plotted.

To quantitate the simultaneous effect of treatment and genotype for each splicing event, we calculated the change in percent spliced in values for the four pairwise comparisons in which the genotype (alternately, treatment) was the same in the pair, but in which the treatment (alternately, genotype) differed. We refer to the unchanged condition as the "context" and to the two conditions that differ as "A" and "B" and denote the corresponding change in the percent spliced in values as $\Delta\Psi_{A-B}^{context} = \Psi_{context,A} - \Psi_{context,B}$. Additionally, we defined the cumulative

effect in a mutant, drug-treated cell relative to a WT, vehicle-treated cell as $\Delta\Psi_{cum} = \Psi_{Sud,MT} - \Psi_{Veh,WT}$. Each of the five comparisons (Veh, WT vs Veh, MUT; Veh, WT vs Sud, WT; Veh, MUT vs Sud, MUT; Sud, WT vs Sud, MUT; Veh, WT vs Sud, MUT) are evaluated using rMATS, with the events subsequently filtered to ensure each isoform was expressed at the level of at least ten sequencing reads in at least as many samples as involved in each of the two conditions compared (i.e., five samples of the ten compared). *p*-values for these events were then adjusted for multiple hypothesis testing as described above. Scatterplots were then plotted of cassette exon skipping events dysregulated by sudemycin in both U2AF1(WT) and U2AF1(S34F) contexts [$|\Delta\Psi^{WT}_{Sud-Veh}|$>1%, $|\Delta\Psi^{MT}_{Sud-Veh}|$>1%, FDR<10% (in both comparisons)].

At the gene-level, PCA was performed as described above for regularized logarithm values for expressed genes. Expressed genes were defined as those with more than 20 counts in at least as many samples as in the smallest condition group, all of which had n=5. Following this minimum read count filtering, genes differentially expressed within the above five conditions were determined via DESeq2.

NanoString assay and analysis

A mouse NanoString probeset was used to validate splice junctions with prior evidence of perturbation by U2AF1(S34F) defined by RNA-seq[12] (unpublished data). A subset of these overlapped events was predicted to be disrupted by sudemycin D6. For each targeted splice junction, a reporter/capture probe pair was designed so that the two probes were complementary to exons on one side or the other of the junction. RNA was prepared using the miRNEasy kit (Qiagen) and samples were processed according to the manufacturer's protocol (NanoString Technologies). RCC files output from the Digital Analyzer were processed using the nSolver Analysis Software to extract raw counts for downstream analysis. Log_2 fold changes

corresponding to the five comparisons above for expressed junctions (i.e., more than 20 reads in at least five samples) were calculated using edgeR.

Supplementary References

1 Griffith, M. *et al.* Genome Modeling System: A Knowledge Management Platform for Genomics. *PLoS computational biology* **11**, e1004274, doi:10.1371/journal.pcbi.1004274 (2015).

2 Kim, D. *et al.* TopHat2: accurate alignment of transcriptomes in the presence of insertions, deletions and gene fusions. *Genome biology* **14**, R36, doi:10.1186/gb-2013-14-4-r36 (2013).

3 Flicek, P. *et al.* Ensembl 2013. *Nucleic acids research* **41**, D48-55, doi:10.1093/nar/gks1236 (2013).

4 Gentleman, R. C. *et al.* Bioconductor: open software development for computational biology and bioinformatics. *Genome biology* **5**, R80, doi:10.1186/gb-2004-5-10-r80 (2004).

5 Anders, S., Pyl, P. T. & Huber, W. HTSeq--a Python framework to work with high-throughput sequencing data. *Bioinformatics* **31**, 166-169, doi:10.1093/bioinformatics/btu638 (2015).

6 Anders, S. & Huber, W. Differential expression analysis for sequence count data. *Genome biology* **11**, R106, doi:10.1186/gb-2010-11-10-r106 (2010).

7 Young, M. D., Wakefield, M. J., Smyth, G. K. & Oshlack, A. Gene ontology analysis for RNA-seq: accounting for selection bias. *Genome biology* **11**, R14, doi:10.1186/gb-2010-11-2-r14 (2010).

8 Shen, S. *et al.* rMATS: robust and flexible detection of differential alternative splicing from replicate RNA-Seq data. *Proceedings of the National Academy of Sciences of the United States of America* **111**, E5593-5601, doi:10.1073/pnas.1419161111 (2014).

9 Soneson, C., Matthes, K. L., Nowicka, M., Law, C. W. & Robinson, M. D. Isoform prefiltering improves performance of count-based methods for analysis of differential transcript usage. *Genome Biol* **17**, 12, doi:10.1186/s13059-015-0862-3 (2016).

10 Benjamini, Y. & Hochberg, Y. Controlling the False Discovery Rate - a Practical and Powerful Approach to Multiple Testing. *Journal of the Royal Statistical Society Series B-Methodological* **57**, 289-300 (1995).

11 Anders, S., Reyes, A. & Huber, W. Detecting differential usage of exons from RNA-seq data. *Genome research* **22**, 2008-2017, doi:10.1101/gr.133744.111 (2012).

12 Shirai, C. L. *et al.* Mutant U2AF1 Expression Alters Hematopoiesis and Pre-mRNA Splicing In Vivo. *Cancer cell* **27**, 631-643, doi:10.1016/j.ccell.2015.04.008 (2015).

13 Ilagan, J. O. *et al.* U2AF1 mutations alter splice site recognition in hematological malignancies. *Genome research*, doi:10.1101/gr.181016.114 (2014).
